# Development of Halide Perovskite Single Crystal for Radiation Detection Applications

**DOI:** 10.3389/fchem.2020.00268

**Published:** 2020-04-23

**Authors:** Wanting Pan, Haotong Wei, Bai Yang

**Affiliations:** ^1^State Key Laboratory of Supramolecular Structure and Materials, College of Chemistry, Jilin University, Changchun, China; ^2^State Key Laboratory of Applied Optics, Changchun Institute of Optics Fine Mechanics and Physics, Chinese Academy of Sciences, Changchun, China

**Keywords:** perovskite, single crystal, synthesis strategies, radiation detection, stability

## Abstract

**Preface:** Recently, low-cost perovskite single crystals have attracted intensive attention due to their excellent optoelectronic properties and improved stability when compared to polycrystalline films for various applications, such as solar cells (Kojima et al., 2009; Lee et al., 2012; Tsai et al., 2016; Sahli et al., 2018), lasers (Gu et al., 2016; Veldhuis et al., 2016), radiation detection (Kim et al., 2017), and so on. The unique optoelectronic properties and low-cost growing processes for large-sized single crystals also make them greatly suitable for radiation detection. In this review, we summarize various synthesis methods of perovskite single crystals and introduced the high radiation detection performance of the perovskite single crystal. The advantages and limitations of halide perovskite single crystals as radiation detector candidates will be discussed in detail, and corresponding future development trends can be expected by overcoming current obstacles (Leijtens et al., 2018; Boyd et al., 2019), such as ion migration (Eames et al., 2015), stability, etc.

## Introduction to Halide Perovskite Single Crystals

Halide perovskite single crystals arouse broad attention, since they can be grown from low-cost solution processes with great potential for future commercialization in optoelectronic devices, especially for radiation detectors. The unique physical and chemical properties of strong stopping power, absence of deep traps, large mu-tau [mobility (μ) and lifetime (τ), μτ] product, and easy crystallization from low-cost solution processes make perovskite single crystals suitable for next-generation ionization detection materials. The high performance and bright future of perovskite single crystals as X-ray imaging detectors, gamma ray detectors, and scintillators will be discussed in the review. While various methods appeared for growing perovskite single crystal, the crystalline processes are in line with traditional crystallization kinetic theory (Zhao et al., 2018; Yin et al., 2019), basically including nucleation and crystal growth. And the nucleation process, which is of paramount importance for the crystal quality, occurs at the beginning of the entire crystalline process. Crystalline speed and static equilibrium stability of saturation solution are the points for growing a high-quality and large-scale perovskite single crystal. As of now, various growth methods for perovskite single crystals with different composition, size, and exposed facets have been already reported. Herein, perovskite single crystals growth methods, including aqueous solution crystallization by cooling method (Yin et al., 2019), traditional high temperature Bridgman method (Stoumpos et al., 2013a), inverse temperature crystallization method (ITC) (Kadro et al., 2015; Saidaminov et al., 2015; Abdelhady et al., 2016; Zhumekenov et al., 2016), and anti-solvent vapor assisted method (ASV) (Shi et al., 2015; Rakita et al., 2016; Wei et al., 2016). The distinguished advantages of these methods will be discussed in detail.

## Single Crystals Growth Strategy

**Figure 1:**
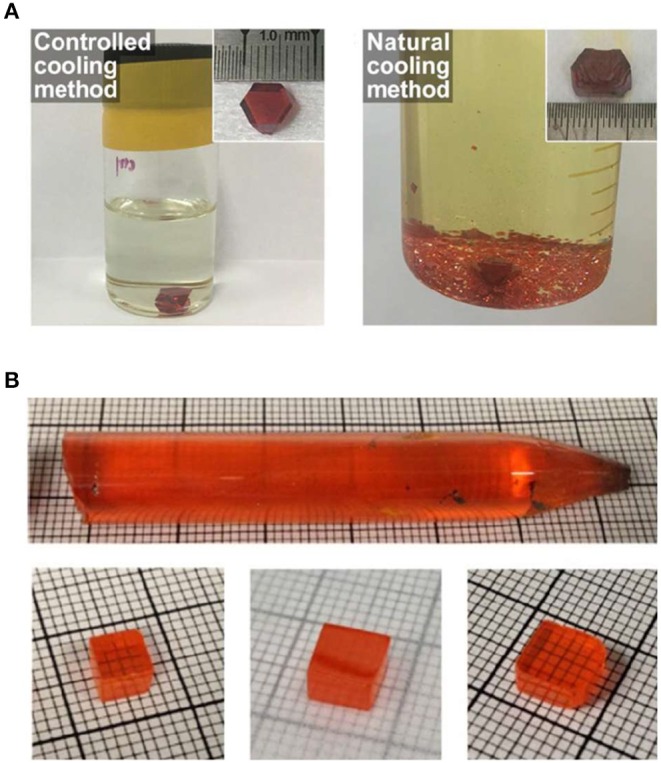
**(A)** The resulting solutions and crystals by controlled (left) and natural (right) cooling methods. The inset photo is the synthesized crystals. **(A)** Reproduced from Yin et al. (2019) with permission from Wiley-VCH. **(B)** Bridgman method grown CsPbBr_3_ single crystal with a diameter of 11 nm, and the single crystal wafer with different sizes. **(B)** Reproduced from He et al. (2018) with permission from the Nature Publishing Group.

Halide perovskite single crystal was first grown by gradually cooling the temperature of saturated solution from aqueous acid solution (Baikie et al., 2013; Stoumpos et al., 2013a; Pisoni et al., 2014; Dang et al., 2015). In the crystallization process, slowing down the cooling rate normally leads to larger single crystals with fewer seed crystals and higher quality single crystal with fewer defects (Figure 1A), which enables larger crystals dimensional sizes, smoother morphology, and a better optoelectronic performance (Yin et al., 2019). Similar to the controlled cooling processes, perovskite single crystals can be also grown from all-solid raw materials by the traditional high temperature Bridgman method (Stoumpos et al., 2013b; He et al., 2018). Despite the long amount of time and high energy consumption involved, raising temperature above solid's melting point is suitable for raw materials with low solubility, like all-inorganic perovskite. This method was always used to grow large-sized CsPbBr_3_ single crystal serving as radiation detection (Stoumpos et al., 2013a; He et al., 2018; Figure 1B). Furthermore, perovskite materials are discovered to represent good solubility in highly polar organic solvent, such as N, N-Dimethylformamide (DMF), Dimethyl sulfoxide (DMSO), etc., and presents an inverse temperature crystallization process, which means lower solubility in higher temperatures within a certain temperature range. This crystallization process was so-called inverse temperature crystallization method (ITC); the crystallization velocity is an order magnitude faster than that of high temperature Bridgman method. ITC method attracted intensive investigations on perovskite crystallization.

**Figure 2:**
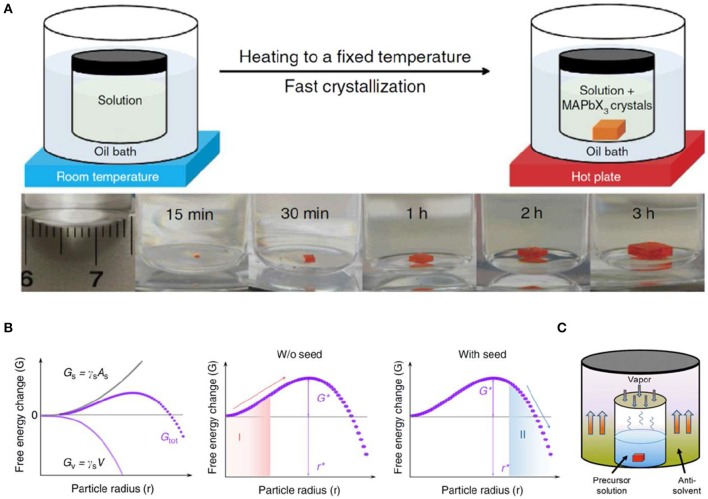
**(A)** Top: schematic representation of the ITC apparatus where the crystallization vial is immersed in a heating bath. Bottom: MAPbBr_3_ crystal growth at different time intervals. **(A)** Reproduced from Saidaminov et al. (2015) with permission from the Nature Publishing Group. **(B)** The Gibbs energy, G_tot_, as a function of particle radius. G_tot_ consists of a volume term, G_v_, and a surface term, G_s_, where γ_s_ and γ_v_ are the surface free energy per unit area and volume free energy per unit volume, respectively (left). Evolution of G_tot_ during the crystallization process without (middle) and with (right) the seed crystal. **(B)** Reproduced from Zhao et al. (2018) with permission from Nature Publishing Group. **(C)** The Schematic diagram of MAPbBr_3_ crystals grown from the solution by the AVC method. **(C)** Reproduced from Liu X. et al. (2018) with permission from the American Chemical Society.

ITC used for crystals with retrograde solubility allows the saturated solution to reach equilibrium and to be filtered at room temperature, as a result, maximizing the operation window, first. Then perovskite single crystals with inverse solubility can be grown at an elevated temperature, and growth velocity is always finely controlled by temperature (Figure 2A). A great deal of perovskite single crystals by ITC method have been reported as exhibiting excellent properties with fast growth velocity, tunable shape, and high optoelectronic performance, when compared with traditional crystal growth methods; and ITC method has been employed for kinds of high-quality perovskite crystals, such as methylammonium lead triiodide (MAPbI_3_), methylammonium lead tribromide (MAPbBr_3_), formamidinium lead triiodide (FAPbI_3_), etc. Figure 2B shows different perovskite crystallization processes in which a crystal seed is added into the saturated precursor solution by ITC method, and the crystal grows at faster crystallization rate, resulting in larger crystal size. This is mainly caused by the critical Gibbs free energy G^*^ required for nucleation (Yang et al., 2015, 2017; Ummadisingu et al., 2017). Thus, when a seed occurs in the precursor solution (region II shown in Figure 2B), it can induce spontaneous crystal growth, shorten growth time, and result in a large-sized single crystal (Saidaminov et al., 2015; Han et al., 2016), otherwise, the crystal will disintegrate spontaneously in region I. Nevertheless, a continuously increased temperature during ITC method will inevitably cause the decomposition of perovskite and reduce the atomic utilization efficiency of raw materials.

Anti-solvent vapor-assisted crystallization (AVC) method can greatly improve the atomic utilization efficiency of raw materials. In this method, anti-solvent will gradually diffuse into perovskite solution and precipitate the crystals (Figure 2C). The crystal growth velocity is determined by the diffusion rate of anti-solvent, as well as the corresponding solubility difference in diverse mixture solvent. Another advantage of AVC method lies in that high-quality crystals can be grown at room temperature with low energy consumption (Rakita et al., 2016; Liu X. et al., 2018), which is more suitable for all-inorganic CsPbX_3_ (X = Cl, Br, I) single crystals when compared to the traditional Bridgman and ITC processes. When mixing equal equivalent CsBr and PbBr_2_ into the solvent, non-stoichiometry crystal nucleus easily appears first in the precursor solution, owing to the mismatched solubility of PbBr_2_ and CsBr. Hence, to get single crystals with stoichiometric ratio, non-stoichiometry crystal nucleus is normally precluded first before entering into the crystal growth process. Then, the rest of the precursor solution with non-equivalent raw materials is filtered to grow crystals with CsPbBr_3_ composition. Moreover, another approach to growing CsPbBr_3_ single crystals is by directly changing the feeding ratio of the raw materials—the optimized ratio for CsBr:PbBr_2_ being 1:1.5 (Zhang et al., 2017). However, the limitation of AVC methods lies in that it is not easily scaled up in industry, and the reproducibility is not as promising as other methods.

Based on these synthesis methods, different kinds of high-quality perovskite single crystals are grown from solution with the aim to understand the physical and chemical properties of perovskite materials for potential optoelectronic applications. Nevertheless, the applications of perovskite materials in optoelectronic devices are mainly focused on the thin film devices rather than single crystal devices, which are basically limited by their thickness and scaling up problem. A thick semiconductor layer often results in a high potential of charge carrier recombination for solar cells devices and a high possibility of reabsorption of the radiative emission from deep inside for light-emitting diodes, since the photo-electric conversions of both these devices are related to the low-energy UV-visible photons. However, perovskite single crystals are actually appealing for high-energy photon detection. In this sense, the thickness of single crystal is an advantage for high attenuation efficiency, and the charges generated inside the crystal can be extracted by applying a bias voltage on the opposite electrodes.

## Halide Perovskite Single Crystal Ionizing Detectors for Imaging

**Figure 3:**
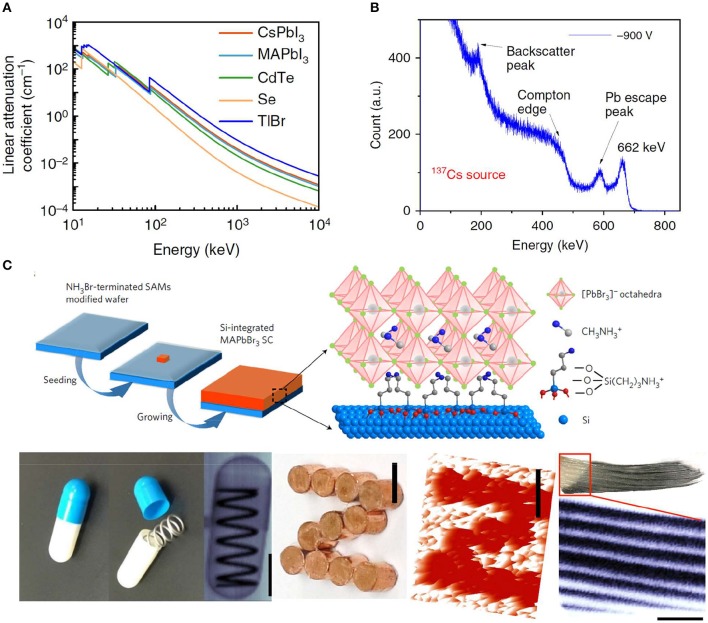
**(A)** Linear attenuation coefficient of MAPbI_3_, CdTe, Se, and T1Br verse photons energy. **(A)** Reproduced from Wei and Huang (2019) with permission from the Nature Publishing Group. **(B)** Energy-resolved spectrum of ^137^Cs γ-ray source with the characteristic energy of 662 keV obtained by a CsPbBr_3_ detector. **(B)** Reproduced from He et al. (2018) with permission from the Nature Publishing Group. **(C)** Schematic illustration of the fabrication Si-integrated MAPbBr_3_ single crystal (top) and optical and X-ray images (bottom). **(C)** Reproduced from Wei W. et al. (2017) with permission from the Nature Publishing Group.

Semiconductor working in direct detection mode normally possesses high sensitivity and high spatial resolution of images. However, there are limited choices of semiconductors for direct detection mode, since it requires semiconductors of high atomic numbers for strong stopping power, large μτ product for high charge collection efficiency, low trap density for less noise and less non-radiative recombination. Halide perovskite single crystal has emerged as a new generation of ionization detection materials working in direct detection mode (Wei et al., 2016; Wei and Huang, 2019), which can directly convert the ionizing charges into readable current by driving the charges with a bias on opposite electrodes. Firstly, perovskite single crystals are easily grown from low-cost solution processes. The estimated price of a perovskite single crystal is about US$ 0.5–1.0 per cm^3^, when combined with the cost of raw materials and fabrication process (Yakunin et al., 2016). Secondly, perovskite single crystal advances in device performance as a candidate of radiation detector in terms of sensitivity, response speed, imaging spatial resolution, etc. The high atomic number of the elements Pb, Cs, Sn, I, and Br and the large density of perovskite result in comparable attenuation coefficient to CdZnTe, which is much higher than α-Se or Si semiconductors shown in Figure 3A (Huang et al., 2019). This is also the reason that Si cannot be employed for high-energy ionization detection material. Once again, the large μτ product and low trap density ensure a high charge collection efficiency under large bias, although there is polarization effect remaining unsolved for halide perovskite materials. Lastly, halide perovskite comprises a large family, including 3D materials with ABX_3_ structure, 2D materials with R_2_*A*_n−1_*B*_n_*X*_3n+1_ structure, and double perovskite with A_2_MM′X_6_ structure. All of the A^+^, B^2+^, M^+^, M^′3+^, and R^+^ ions are variable by following the Goldschmidt tolerance factor principle to stabilize the perovskite phase (Goldschmidt, 1926), which is closely related to the ion's radius. A large variety of chemical and physical properties can be obtained from perovskite by fine-tuning the ions and structures.

MAPbBr_3_ single crystal has been demonstrated to be a promising X-ray imager, exhibiting excellent performance (Yakunin et al., 2015; Wei W. et al., 2017; Liu Y. et al., 2018). In addition, MAPbBr_3_ single crystal was also successfully integrated on Si devices showing a sensitivity to X-rays over 1,000 times greater than that of α-Se detectors and an ability to image at a 15 to 120-fold weaker X-ray flux, as depicted in Figure 3C. The device can detect a very low dose rate of <0.1 μGy_air_s^−1^ with a high sensitivity of 2.1 × 10^4^ μC ${\text{Gy}}_{\text{air}}^{-1}$cm^−2^ to 8 keV X-rays, providing a strategy to obtain active-matrix flat-panel imagers for further commercialization application as X-ray detector in medical and security check (Wei W. et al., 2017). Also, lead-free perovskite single crystals have developed rapidly in recent years, and Cs_2_AgBiBr_6_ single crystal detector has realized a minimum detectable dose rate as low as 59.7 nGy_air_s^−1^, comparable to the reported MAPbBr_3_ (Pan et al., 2017). And, more stable 2D lead-free double perovskite single crystals of (BA)_2_CsBiBr_7_ and (NH_4_)_3_Bi_2_I_9_ have minimum detectable dose rate of 4.5 μGy_air_s^−1^ and 55 nGy_air_s^−1^, respectively, which are also comparable to that of MAPbBr_3_ single crystal (Xu et al., 2019; Zhuang et al., 2019).

## Halide Perovskite Single Crystal Gamma Ray Detectors for Energy Spectroscopy

The decay of the majority of radioactive isotopes involves the irradiation of gamma (γ) photons with energies of ~200 keV to 10 MeV. Low-cost and highly sensitive detectors, which can operate at ambient temperature, are of critical importance for many applications, such as homeland defense, nuclear reaction inspection, research field, etc. (Milbrath et al., 2008; Sordo et al., 2009). Perovskite semiconductor is one type of promising radiation detection candidates with μτ product of 1 × 10^−2^ cm^2^ V^−1^, stopping power, and bulk resistivity comparable or even better than state-of-the-art detector material Cd_1−x_Zn_x_Te (*x* <20%, denoted as CZT) or thallium (I) bromide (TlBr) devices. Furthermore, halide perovskite crystals can be grown from low-cost solution processes based on earth-abundant raw materials, which is more suitable for industrial production when compared to the high-temperature Bridgeman methods. Perovskite single crystals with various composition (MAPbI_3_, FAPbI_3_, CsPbBr_3_, I-treated/Cl-treated MAPbBr_3_) were applied to serve as solid-state gamma-detecting materials with comparable energy resolution to standard scintillator detectors (Yakunin et al., 2016; Wei H. et al., 2017; He et al., 2018; Wei and Huang, 2019), owing to their superior device signal/noise ratio, which is benefited from a large μτ product of 1.0–1.8 × 10^−2^ cm^2^ V^−1^ (Yakunin et al., 2016; Wei H. et al., 2017) and a low dark current and noise. All of these enable a promising device response to 59.5 keV ^241^Am γ-source, 122 keV ^57^Co source, and 662 keV ^137^Cs source with a sufficient energy resolution of 3.9% (Figure 3B), which is comparable to the commercial NaI (Tl) scintillator. However, the ion migration phenomenon can be still observed in these detection devices, resulting in continuous dark current drift and degraded energy spectrum resolution (Luo et al., 2017).

## Perovskite Single Crystals as Scintillator

**Figure 4:**
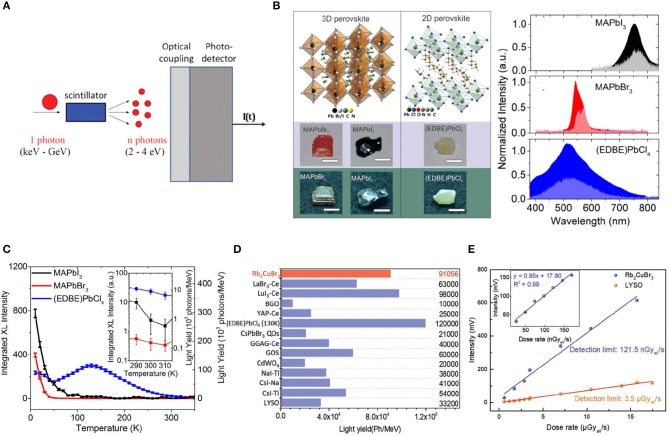
**(A)** Scintillator working principal and set-up of a scintillation detector. **(A)** Reproduced from Nikl and Yoshikawa (2015) with permission from Wiley-VCH. **(B)** Crystal structure and appearance and emission spectra under X-ray (left) and optical excitation (right). **(C)** Temperature dependence of the light yields. **(B,C)** Reproduced from Birowosuto et al. (2016) with permission from the Nature Publishing Group. **(D)** The comparison of light yield between Rb_2_CuBr_3_ and several well-known scintillators. **(E)** Emission intensity of Rb_2_CuBr_3_ and LYSO as a linear function to dose rate. The inset is the data of Rb_2_CuBr_3_ below 200 nGy_air_/s for detection limit measurement. **(D,E)** Reproduced from Yang et al. (2019) with permission from Wiley-VCH.

Scintillator is another kind of ionizing detector working in indirect detection mode, which converts high-energy X-ray photons to low-energy UV-visible photons upon irradiation. A sensitive photodetector is integrated with the scintillator to detect the emitting UV-visible photons (Figure 4A). Therefore, it is important for a scintillator to have: (1) thick single crystal with strong stopping power to fully attenuate the high-energy photons, and transparent enough to emit light with less scattering; (2) large light emission yield to convert the irradiation energy to detectable energy as much as possible; (3) large stokes shift to avoid reabsorption of the emission from the thick scintillator crystals; and (4) fast radiation recombination speed to reduce the response time. In order to satisfy these requirements, scintillator can be composed of 2D materials or quantum dots materials to confine the ionizing charges around a local area to increase the radiative recombination velocity (Zhou et al., 2017, 2018). Foreign ions are often doped into the bulk single crystals to act as radiative emission center, which is a general strategy to enlarge the stokes shift and increase the light emission yield (Cavouras et al., 1996; Uchiyama et al., 2001).

MAPbI_3_, MAPbBr_3_, and 2D (EDBE)_2_PbCl_4_ [EDBE = 2,2′-(ethylenedioxy)bis(ethylammoniu-m)] (Figure 4B) single crystals have been studied for scintillator application, and photoluminescence (PL) peak of MAPbI_3_ single crystal is centered at 780 nm. Meanwhile, PL peaks of MAPbBr_3_ and 2D (EDBE)PbCl_4_ locate at 550 and 520 nm, respectively (Figure 4B), which can match the common sensitive photodetector (Birowosuto et al., 2016). The bandgap emission of perovskite single crystal scintillator can be easily optimized through engineering of the composition. 2D perovskite structure with long chain organic spacer shows higher light yield (Li et al., 2019; Figure 4C). Recently, Rb_2_CuBr_3_ perovskite single crystal showed promising scintillator properties with a light yield of over 9 × 10^4^ Photons/MeV, higher than traditional bright scintillators of CsI:Tl (5.4 × 10^4^ Photons/MeV) and alginate-derived guluronate oligosaccharide (GOS) (6.0 × 10^4^ Photons-/MeV) (Figure 4D), and reaching a low X-ray detection limit of 121.5 nGy_air_s^−1^ (Yang et al., 2019; Figure 4E).

Similar to the semiconductor in direct detection mode, scintillator single crystals also need a low trap density to avoid the non-radiative recombination in the scintillator material, which often leads to a slow components in scintillation decay and afterglow. Therefore, low trap density single crystals are also necessary for high-performance ionization detection. However, the fluorescence from emission center in single crystal is isotropic, which normally ruins the detectable light emitting yield when there is an absence of an integrating sphere and also results in a low spatial resolution by mutual interference with neighboring pixels on a flat panel (Grim et al., 2012; Heo et al., 2018; Burdette et al., 2019).

## Improvement Strategies for Better Device Performance

**Figure 5:**
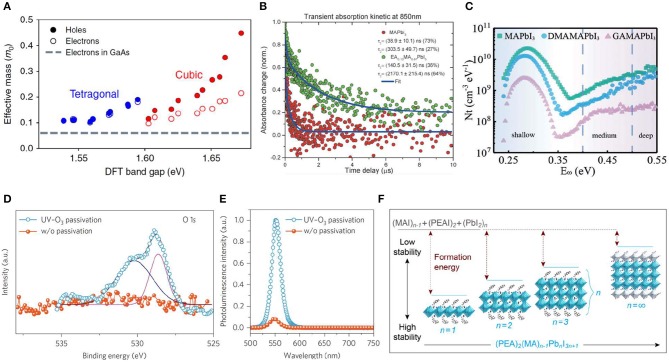
**(A)** Carrier effective masses. Effective masses of electrons and holes in the cubic (red) and tetragonal (blue) MAPbI_3_ phases vs. the band gap, shown for 10 structures (in each phase) that differ only in their MA random alignment. The dotted line given for comparison represents the electron effective mass of GaAs. The LDA band gaps were shifted by ~1.2 eV to match the experimental gap. **(A)** Reproduced from Frohna et al. (2018) with permission from the Nature Publishing Group. **(B)** TA kinetics of MAPbI_3_ and EA_0.17_MA_0.83_PbI_3_ single crystals. The time constants of the fitting are listed. **(B)** Reproduced from Peng et al. (2016) with permission from Wiley-VCH. **(C)** Thermal admittance spectroscopy (TAS) spectrum of MAPbI_3_, DMAPbI_3_, and GAMAPbI_3_. **(C)** Reproduced from Huang et al. (2019) with permission from Wiley-VCH. **(D)** XPS spectra of the MAPbBr_3_ thin film for O 1s before and after UV-O_3_ passivation. **(E)** Photoluminescence spectra of a MAPbBr_3_ single crystal in vacuum before and after UV-O_3_ treatment. **(D,E)** Reproduced from Wei et al. (2016) with permission from the Nature Publishing Group. **(F)** Schematic diagram of dimension tailoring and stability. **(F)** Reproduced from Quan et al. (2016) with permission from the American Chemical Society.

Defects, which exist at both surface and bulk of perovskite single crystal, are detrimental for improving device performance in most cases despite absence of deep traps in halide perovskite materials (Shao et al., 2014; Wang et al., 2018; Chen et al., 2019). Passivating the defects in perovskite is a necessary requirement for high-performance devices. MAPbI_3_, as a typical material in the halide perovskite family, is still rich of shallow defects such as Pb^2+^ interstitial and I^−^ vacancy, and so on. To reduce the intrinsic bulk defects in MAPbI_3_ single crystal, large-sized A site cation was doped inside to tune tetragonal phase to more stable cubic phase (Figure 5A) by balancing opposite lattice distortion strains (Mitzi, 2001; Stoumpos et al., 2013b; Peng et al., 2016). Furthermore, long-chain organic cations as A site can result in the so-called quasi-2D perovskite structure, which is more ambient stable compared to the 3D structure halide perovskite (Peng et al., 2016). Moreover, 2D structure can also decrease electron–phonon coupling strength and increase material defects formation energy (Huang et al., 2019). Composition engineering, especially introducing large-sized cations of EA (ethylammonium), DMA (dimethylammonium), and GA (guaniddinium), can obviously increase the charge carrier lifetime and decrease the defect density (Figures 5B,C), i.e., increased μτ product (Peng et al., 2016; Huang et al., 2019), which is of great significance for radiation detectors. In addition to intrinsic bulk defects, crystal surface is also rich in shallow traps. UV-O_3_ treatment was employed to passivate the defects on MAPbBr_3_ single crystal top surface (Wei et al., 2016), revealing the Pb^2+^ dangling bonds passivated by forming oxygen–lead bonds after UV-O_3_ treatment (Figures 5D,E).

Another obstacle that should be faced for perovskite X-ray detector is the ions migration phenomenon, which normally results in the polarization effect in ionization detection (Eames et al., 2015; Luo et al., 2017; Wei and Huang, 2019). Suppressing the ions migration is challenging in perovskite ionizing detector since a large bias is often applied, and the ionic intrinsic properties of perovskite determine the low activation energy of mobile ions. One strategy to suppress the ions migration is to block the ion mobile path and elevate the corresponding activation energy. Low dimensional perovskite single crystal has long-term stability under operation condition (Smith et al., 2014; Cao et al., 2015; Tsai et al., 2016; Yuan et al., 2016), and the generic structural formula of 2D perovskite is A_2_*B*_n−1_*M*_n_*X*_3n+1_ (n is integer), A^+^ is a primary aliphatic or aromatic alkylammonium cation, M^2+^ is a divalent metal, and X^−^ is a halide anion. [MX_6_]^4−^ forms octahedra frame structure, suitably sized B^+^ cation insert into the octahedra lattice, and bulky A^+^ cations form an organic spacer existing between the semiconducting inorganic layers, which can effectively suppress the ions migration (depicted in Figure 5F).

## Summary and Future Prospects

As one of the high-performance semiconductors, perovskite is proposed to forge a bright future in the radiation detection field for its earth-abundant raw materials, low cost, feasible fabrication, and high-performance characteristics. However, the semiconductor's long-term instability limits its future application in many fields. To improve the long-term stability, researchers have reported various improvement strategies. 1, tuning the ratio of halide ions, such as chlorine incorporation into MAPbBr_3_ and bromine incorporation into MAPbI_3_, this can obviously improve the moisture stability of the perovskite (Noh et al., 2013; Wei H. et al., 2017; Boyd et al., 2019; Hieulle et al., 2019). 2, changing the halide into halide substitution, like totally or partly replacing halide with thiocyanate (SCN) ion (Jiang et al., 2015). 3, doping additives (i.e. quantum dots, polymer material and small organic molecules) in precursor (Wang et al., 2017; Hieulle et al., 2019; Yao et al., 2019). What‘s more, 2D perovskites [(A)_2_(B)_n−1_M_n_X_3n+1_ (n is integer)] have emerged as a more moisture stable material, and researchers designed more inter-molecular interactions in 2D perovskites by tunneling the A-site molecule, which have further improved the stability. On the other hand, researchers have reported lead-free perovskite as non-toxic substitutes, but the performance is still limited, so an urgent objective is to improve the performance of 2D perovskite and lead-free perovskite devices. Obtaining high-quality perovskite crystal materials is crucial for better device performance and stability, and the means of attaining a perovskite crystal with fewer defects and of a larger size remains obviously unsolved. More efforts are still needed to improve the synthesis techniques of the perovskite single crystals and to boost the performance of promising ionization detection applications.

## Author Contributions

HW and WP wrote the manuscript. All authors contributed to the commenting and review of the manuscript.

## Conflict of Interest

The authors declare that the research was conducted in the absence of any commercial or financial relationships that could be construed as a potential conflict of interest.

## References

[B1] Abdelhady A. L. Saidaminov M. I. Murali B. Adinolfi V. Voznyy O. Katsiev K.. (2016). Heterovalent dopant incorporation for bandgap and type engineering of perovskite crystals. J. Phys. Chem. Lett. 7, 295–301. 10.1021/acs.jpclett.5b02681 26727130

[B2] Baikie T. Fang Y. Kadro J. M. Schreyer M. Wei F. Mhaisalkar S. G. (2013). Synthesis and crystal chemistry of the hybrid perovskite (CH_3_NH_3_)PbI_3_ for solid-state sensitised solar cell applications. J. Mater. Chem. A 1, 5628–5641. 10.1039/c3ta10518k

[B3] Birowosuto M. D. Cortecchia D. Drozdowski W. Brylew K. Lachmanski W. Bruno A.. (2016). X-ray scintillation in lead halide perovskite crystals. Sci. Rep. 6:37254. 10.1038/srep37254 27849019 PMC5111063

[B4] Boyd C. C. Cheacharoen R. Leijtens T. McGehee M. D. (2019). Understanding degradation mechanisms and improving stability of perovskite photovoltaics. Chem. Rev. 119, 3418–3451. 10.1021/acs.chemrev.8b00336 30444609

[B5] Burdette M. K. Bandera Y. P. Zhang E. Trofimov A. Dickey A. Foulger I.. (2019). Organic fluorophore coated polycrystalline ceramic LSO:Ce scintillators for X-ray bioimaging. Langmuir 35, 171–182. 10.1021/acs.langmuir.8b03129 30518207

[B6] Cao D. H. Stoumpos C. C. Farha O. K. Hupp J. T. Kanatzidis M. G. (2015). 2D homologous perovskites as light-absorbing materials for solar cell applications. J. Am. Chem. Soc. 137, 7843–7850. 10.1021/jacs.5b03796 26020457

[B7] Cavouras D. Kandarakis I. Panayiotakis G. S. Evangelou E. K. Nomicos C. D. (1996). An evaluation of the Y_2_O_3_:Eu^3+^ scintillator for application in medical x-ray detectors and image receptors. Med. Phys. 23, 1965–1975. 10.1118/1.597769 8994161

[B8] Chen Y. Li N. Wang L. Li L. Xu Z. Jiao H.. (2019). Impacts of alkaline on the defects property and crystallization kinetics in perovskite solar cells. Nat. Commun. 10:1112. 10.1038/s41467-019-09093-1 30846692 PMC6405758

[B9] Dang Y. Liu Y. Sun Y. Yuan D. Liu X. Lu W. (2015). Bulk crystal growth of hybrid perovskite material CH_3_NH_3_PbI_3_. CrystEngComm 17, 665–670. 10.1039/C4CE02106A

[B10] Eames C. Frost J. M. Barnes P. R. O'Regan B. C. Walsh A. Islam M. S. (2015). Ionic transport in hybrid lead iodide perovskite solar cells. Nat. Commun. 6:7497. 10.1038/ncomms8497 26105623 PMC4491179

[B11] Frohna K. Deshpande T. Harter J. Peng W. Barker B. A. Neaton J. B.. (2018). Inversion symmetry and bulk Rashba effect in methylammonium lead iodide perovskite single crystals. Nat. Commun. 9:1829. 10.1038/s41467-018-04212-w 29739939 PMC5940805

[B12] Goldschmidt V. M. (1926). Die Gesetze der Krystallochemie. Naturwissenschaften 14, 477–485. 10.1007/BF01507527

[B13] Grim J. Q. Li Q. Ucer K. B. Burger A. Bizarri G. A. Moses W. W. (2012). The roles of thermalized and hot carrier diffusion in determining light yield and proportionality of scintillators. Physica Status Solidi (a) 209, 2421–2426. 10.1002/pssa.201200436

[B14] Gu Z. Wang K. Sun W. Li J. Liu S. Song Q. (2016). Two-photon pumped CH_3_NH_3_PbBr_3_ perovskite microwire lasers. Adv. Optic. Mater. 4, 472–479. 10.1002/adom.201500597

[B15] Han Q. Bae S. H. Sun P. Hsieh Y. T. Yang Y. M. Rim Y. S.. (2016). Single crystal formamidinium lead iodide (FAPbI_3_): insight into the structural, optical, and electrical properties. Adv. Mater. 28, 2253–2258. 10.1002/adma.201505002 26790006

[B16] He Y. Matei L. Jung H. J. McCall K. M. Chen M. Stoumpos C. C.. (2018). High spectral resolution of gamma-rays at room temperature by perovskite CsPbBr_3_ single crystals. Nat. Commun. 9:1609. 10.1038/s41467-018-04073-3 29686385 PMC5913317

[B17] Heo J. H. Shin D. H. Park J. K. Kim D. H. Lee S. J. Im S. H. (2018). High-performance next-generation perovskite nanocrystal scintillator for nondestructive X-ray imaging. Adv. Mater. 30:1801743 10.1002/adma.201801743 30141200

[B18] Hieulle J. Wang X. Stecker C. Son D. Y. Qiu L. Ohmann R.. (2019). Unraveling the impact of halide mixing on perovskite stability. J. Am. Chem. Soc. 141, 3515–3523. 10.1021/jacs.8b11210 30646682 PMC7156144

[B19] Huang Y. Qiao L. Jiang Y. He T. Long R. Yang F.. (2019). A-site cation engineering for highly efficient MAPbI_3_ single-crystal X-ray detector. Angew. Chem. Int. Edn. 58, 17834–17842. 10.1002/anie.201911281 31549478

[B20] Jiang Q. Rebollar D. Gong J. Piacentino E. L. Zheng C. Xu T. (2015). Pseudohalide-induced moisture tolerance in perovskite CH_3_NH_3_Pb(SCN)_2_I thin films. Angew. Chem. 127, 7727–7730. 10.1002/ange.201503038 25968343

[B21] Kadro J. M. Nonomura K. Gachet D. Grätzel M. Hagfeldt A. (2015). Facile route to freestanding CH_3_NH_3_PbI_3_ crystals using inverse solubility. Sci. Rep. 5:11654. 10.1038/srep11654 26123285 PMC4650687

[B22] Kim Y. C. Kim K. H. Son D. Y. Jeong D. N. Seo J. Y. Choi Y. S.. (2017). Printable organometallic perovskite enables large-area, low-dose X-ray imaging. Nature 550, 87–91. 10.1038/nature24032 28980632

[B23] Kojima A. Teshima K. Shirai Y. Miyasaka T. (2009). Organometal halide perovskites as visible-light sensitizers for photovoltaic cells. J. Am. Chem. Soc. 131, 6050–6051. 10.1021/ja809598r 19366264

[B24] Lee M. M. Teuscher J. Miyasaka T. Murakami T. N. Snaith H. J. (2012). Efficient hybrid solar cells based on meso-superstructured organometal halide perovskites. Science 338, 643–647. 10.1126/science.1228604 23042296

[B25] Leijtens T. Bush K. A. Prasanna R. McGehee M. D. (2018). Opportunities and challenges for tandem solar cells using metal halide perovskite semiconductors. Nat. Energy 3, 828–838. 10.1038/s41560-018-0190-4

[B26] Li Y. Shao W. Ouyang X. Zhu Z. Zhang H. Ouyang X. (2019). Scintillation properties of perovskite single crystals. J. Phys. Chem. C 123, 17449–17453. 10.1021/acs.jpcc.9b05269

[B27] Liu X. Zhang H. Zhang B. Dong J. Jie W. Xu Y. (2018). Charge transport behavior in solution-grown methylammonium lead tribromide perovskite single crystal using α particles. J. Phys. Chem. C 122, 14355–14361. 10.1021/acs.jpcc.8b03512

[B28] Liu Y. Zhang Y. Zhao K. Yang Z. Feng J. Zhang X. (2018). A 1300 mm^2^ ultrahigh-performance digital imaging assembly using high-quality perovskite single crystals. Adv. Mater. 30:1707314 10.1002/adma.201707314 29845652

[B29] Luo Y. Khoram P. Brittman S. Zhu Z. Lai B. Ong S. P.. (2017). Direct observation of halide migration and its effect on the photoluminescence of methylammonium lead bromide perovskite single crystals. Adv. Mater. 29:1703451. 10.1002/adma.201703451 28961331

[B30] Milbrath B. D. Peurrung A. J. Bliss M. Weber W. J. (2008). Radiation detector materials: an overview. J. Mater. Res 23, 2561–2581. 10.1557/JMR.2008.0319

[B31] Mitzi D. B. (2001). Templating and structural engineering in organic–inorganic perovskites. J. Chem. Soc. Dalton Trans. 2001, 1–12. 10.1039/b007070j

[B32] Nikl M. Yoshikawa A. (2015). Recent RandD trends in inorganic single-crystal scintillator materials for radiation detection. Adv. Optic. Mater. 3, 463–481. 10.1002/adom.201400571

[B33] Noh J. H. Im S. H. Heo J. H. Mandal T. N. Seok S. I.. (2013). Chemical management for colorful, efficient, and stable inorganic–organic hybrid nanostructured solar cells. Nano Lett. 13, 1764–1769. 10.1021/nl400349b 23517331

[B34] Pan W. Wu H. Luo J. Deng Z. Ge C. Chen C. (2017). Cs_2_AgBiBr_6_ single-crystal X-ray detectors with a low detection limit. Nat. Photonics 11, 726–732. 10.1038/s41566-017-0012-4

[B35] Peng W. Miao X. Adinolfi V. Alarousu E. Tall O. E. Emwas A. H. (2016). Engineering of CH_3_NH_3_PbI_3_ perovskite crystals by alloying large organic cations for enhanced thermal stability and transport properties. Angew. Chem. Int. Edn. 55, 10686–10690. 10.1002/anie.201604880 27468159

[B36] Pisoni A. Jaćimović J. Barišić O. S. Spina M. Gaál R. Forró L.. (2014). Ultra-low thermal conductivity in organic–inorganic hybrid perovskite CH_3_NH_3_PbI_3_. J. Phys. Chem. Lett. 5, 2488–2492. 10.1021/jz5012109 26277821

[B37] Quan L. N. Yuan M. Comin R. Voznyy O. Beauregard E. M. Hoogland S.. (2016). Ligand-stabilized reduced-dimensionality perovskites. J. Am. Chem. Soc. 138, 2649–2655. 10.1021/jacs.5b11740 26841130

[B38] Rakita Y. Kedem N. Gupta S. Sadhanala A. Kalchenko V. Böhm M. L. (2016). Low-temperature solution-grown CsPbBr_3_ single crystals and their characterization. Cryst. Growth Des. 16, 5717–5725. 10.1021/acs.cgd.6b00764

[B39] Sahli F. Werner J. Kamino B. A. Bräuninger M. Monnard R. Paviet-Salomon B.. (2018). Fully textured monolithic perovskite/silicon tandem solar cells with 25.2% power conversion efficiency. Nat. Mater. 17, 820–826. 10.1038/s41563-018-0115-4 29891887

[B40] Saidaminov M. I. Abdelhady A. L. Murali B. Alarousu E. Burlakov V. M. Peng W.. (2015). High-quality bulk hybrid perovskite single crystals within minutes by inverse temperature crystallization. Nat. Commun. 6:7586. 10.1038/ncomms8586 26145157 PMC4544059

[B41] Shao Y. Xiao Z. Bi C. Yuan Y. Huang J. (2014). Origin and elimination of photocurrent hysteresis by fullerene passivation in CH_3_NH_3_PbI_3_ planar heterojunction solar cells. Nat. Commun. 5:5784. 10.1038/ncomms6784 25503258

[B42] Shi D. Adinolfi V. Comin R. Yuan M. Alarousu E. Buin A.. (2015). Low trap-state density and long carrier diffusion in organolead trihalide perovskite single crystals. Science 347, 519. 10.1126/science.aaa2725 25635092

[B43] Smith I. C. Hoke E. T. Solis-Ibarra D. McGehee M. D. Karunadasa H. I. (2014). A layered hybrid perovskite solar-cell absorber with enhanced moisture stability. Angew. Chem. Int. Edn. 53, 11232–11235. 10.1002/anie.201406466 25196933

[B44] Sordo S. D. Abbene L. Caroli E. Mancini A. M. Zappettini A. Ubertini P. (2009). Progress in the development of CdTe and CdZnTe semiconductor radiation detectors for astrophysical and medical applications. Sensors 9, 3491–3526. 10.3390/s90503491 22412323 PMC3297127

[B45] Stoumpos C. C. Malliakas C. D. Kanatzidis M. G.. (2013b). Semiconducting tin and lead iodide perovskites with organic cations: phase transitions, high mobilities, and near-infrared photoluminescent properties. Inorg. Chem. 52, 9019–9038. 10.1021/ic401215x 23834108

[B46] Stoumpos C. C. Malliakas C. D. Peters J. A. Liu Z. Sebastian M. Im J. (2013a). Crystal growth of the perovskite semiconductor CsPbBr_3_: a new material for high-energy radiation detection. Cryst. Growth Des. 13, 2722–2727. 10.1021/cg400645t

[B47] Tsai H. Nie W. Blancon J. C. Stoumpos C. C. Asadpour R. Harutyunyan B.. (2016). High-efficiency two-dimensional Ruddlesden–Popper perovskite solar cells. Nature 536, 312–316. 10.1038/nature18306 27383783

[B48] Uchiyama Y. Kouda M. Tanihata C. Isobe N. Takahashi T. Murakami T. (2001). Study of energy response of Gd_2_SiO_5_: Ce^3+^scintillator for the ASTRO-E hard X-ray detector. IEEE Trans. Nucl. Sci. 48, 379–384. 10.1109/23.940084

[B49] Ummadisingu A. Steier L. Seo J. Y. Matsui T. Abate A. Tress W.. (2017). The effect of illumination on the formation of metal halide perovskite films. Nature 545, 208–212. 10.1038/nature22072 28445459

[B50] Veldhuis S. A. Boix P. P. Yantara N. Li M. Sum T. C. Mathews N.. (2016). Perovskite materials for light-emitting diodes and lasers. Adv. Mater. 28, 6804–6834. 10.1002/adma.201600669 27214091

[B51] Wang F. Bai S. Tress W. Hagfeldt A. Gao F. (2018). Defects engineering for high-performance perovskite solar cells. NPJ Flexible Electronics 2:22 10.1038/s41528-018-0035-z

[B52] Wang Q. Zhang X. Jin Z. Zhang J. Gao Z. Li Y. (2017). Energy-down-shift CsPbCl_3_:mn quantum dots for boosting the efficiency and stability of perovskite solar cells. ACS Energy Lett. 2, 1479–1486. 10.1021/acsenergylett.7b00375

[B53] Wei H. Bai S. Tress W. Hagfeldt A. Gao F. (2016). Sensitive X-ray detectors made of methylammonium lead tribromide perovskite single crystals. Nat. Photonics 10, 333–339. 10.1038/nphoton.2016.41

[B54] Wei H. DeSantis D. Wei W. Deng Y. Guo D. Savenije T. J.. (2017). Dopant compensation in alloyed CH_3_NH_3_PbBr_3−x_Cl_x_ perovskite single crystals for gamma-ray spectroscopy. Nat. Mater. 16, 826–833. 10.1038/nmat4927 28671663

[B55] Wei H. Huang J. (2019). Halide lead perovskites for ionizing radiation detection. Nat. Commun. 10:1066. 10.1038/s41467-019-08981-w 30842411 PMC6403296

[B56] Wei W. Zhang Y. Xu Q. Wei H. Fang Y. Wang Q. (2017). Monolithic integration of hybrid perovskite single crystals with heterogenous substrate for highly sensitive X-ray imaging. Nat. Photonics 11, 315–321. 10.1038/nphoton.2017.43

[B57] Xu Z. Liu X. Li Y. Liu X. Yang T. Ji C.. (2019). Exploring lead-free hybrid double perovskite crystals of (BA)_2_CsAgBiBr_7_ with large mobility-lifetime product toward X-ray detection. Angew. Chem. Int. Edn. 58, 15757–15761. 10.1002/anie.201909815 31454142

[B58] Yakunin S. Dirin D. N. Shynkarenko Y. Morad V. Cherniukh I. Nazarenko O. (2016). Detection of gamma photons using solution-grown single crystals of hybrid lead halide perovskites. Nat. Photonics 10, 585–589. 10.1038/nphoton.2016.139

[B59] Yakunin S. Sytnyk M. Kriegner D. Shrestha S. Richter M. Matt G. J. (2015). Detection of X-ray photons by solution-processed lead halide perovskites. Nat. Photonics 9, 444–449. 10.1038/nphoton.2015.82 28553368 PMC5444515

[B60] Yang B. Yin L. Niu G. Yuan J. H. Xue K. H. Tan Z. Yang B.. (2019). Lead-free halide Rb_2_CuBr_3_ as sensitive X-ray scintillator. Adv. Mater. 31, 1904711. 10.1002/adma.201904711 31531905

[B61] Yang W. S. Noh J. H. Jeon N. J. Kim Y. C. Ryu S. Seo J.. (2015). High-performance photovoltaic perovskite layers fabricated through intramolecular exchange. Science 348, 1234–1237. 10.1126/science.aaa9272 25999372

[B62] Yang W. S. Park B. W. Jung E. H. Jeon N. J. Kim Y. C. Lee D. U.. (2017). Iodide management in formamidinium-lead-halide–based perovskite layers for efficient solar cells. Science 356, 1376–1379. 10.1126/science.aan2301 28663498

[B63] Yao D. Zhang C. Zhang S. Yang Y. Du A. Waclawik E. Yao D.. (2019). 2D−3D mixed organic–inorganic perovskite layers for solar cells with enhanced efficiency and stability induced by n-propylammonium iodide additives. ACS Appl. Mater. Interfaces 11, 29753–29764. 10.1021/acsami.9b06305 31135124

[B64] Yin L. Wu H. Pan W. Yang B. Li P. Luo J. (2019). Controlled cooling for synthesis of Cs_2_AgBiBr_6_ single crystals and its application for X-ray detection. Adv. Optic. Mater. 7:1900491 10.1002/adom.201900491

[B65] Yuan M. Quan L. N. Comin R. Walters G. Sabatini R. Voznyy O.. (2016). Perovskite energy funnels for efficient light-emitting diodes. Nat. Nanotechnol. 11, 872–877. 10.1038/nnano.2016.110 27347835

[B66] Zhang H. Liu X. Dong J. Yu H. Zhou C. Zhang B. (2017). Centimeter-sized inorganic lead halide perovskite CsPbBr_3_ crystals grown by an improved solution method. Cryst. Growth Des. 17, 6426–6431. 10.1021/acs.cgd.7b01086

[B67] Zhao Y. Tan H. Yuan H. Yang Z. Fan J. Z. Kim J.. (2018). Perovskite seeding growth of formamidinium-lead-iodide-based perovskites for efficient and stable solar cells. Nat. Commun. 9:1607. 10.1038/s41467-018-04029-7 29686304 PMC5913260

[B68] Zhou C. Lin H. Tian Y. Yuan Z. Clark R. Chen B.. (2018). Luminescent zero-dimensional organic metal halide hybrids with near-unity quantum efficiency. Chem. Sci. 9, 586–593. 10.1039/c7sc04539e 29629122 PMC5870054

[B69] Zhou C. Tian Y. Wang M. Rose A. Besara T. Doyle N. K.. (2017). Low-dimensional organic tin bromide perovskites and their photoinduced structural transformation. Angew. Chem. Int. Edn. 56, 9018–9022. 10.1002/anie.201702825 28573667

[B70] Zhuang R. Wang X. Ma W. Wu Y. Chen X. Tang L. (2019). Highly sensitive X-ray detector made of layered perovskite-like (NH_4_)_3_Bi_2_I_9_ single crystal with anisotropic response. Nat. Photonics 13, 602–608. 10.1038/s41566-019-0466-7

[B71] Zhumekenov A. A. Saidaminov M. I. Haque M. A. Alarousu E. Sarmah S. P. Murali B. (2016). Formamidinium lead halide perovskite crystals with unprecedented long carrier dynamics and diffusion length. ACS Energy Lett. 1, 32–37. 10.1021/acsenergylett.6b00002

